# Pregnane X receptor suppresses proliferation and tumourigenicity of colon cancer cells

**DOI:** 10.1038/sj.bjc.6605677

**Published:** 2010-06-08

**Authors:** N Ouyang, S Ke, N Eagleton, Y Xie, G Chen, B Laffins, H Yao, B Zhou, Y Tian

**Affiliations:** 1 Department of Veterinary Physiology and Pharmacology, Texas A&M University, College Station, TX 77843, USA; 2 The First Affiliated Hospital of Guangzhou Medical College, Guangzhou, China; 3 Chemotherapy Department, Sun Yet-Sen Memorial Hospital, Guangzhou, China

**Keywords:** pregnane X receptor, colon cancer, Ki-67 antigen, E2F1 protein

## Abstract

**Background:**

Pregnane X receptor (PXR) is a nuclear receptor that regulates the metabolism and disposition of various xenobiotics and endobioitics. We investigated a novel PXR function in regulating colon tumourigenesis in this study.

**Methods:**

Histochemistry, transfection, cell proliferation assay, anchorage-*α*-dependent assay, xenograft, immunohistochemistry, immunofluorescence flow cytometry.

**Results:**

Using histochemistry analysis, we found that PXR expressions were lost or greatly diminished in many colon tumours. Ectopic expression of human PXR through stable transfection of PXR into colon cancer cell line HT29 significantly inhibited cell proliferation as determined by cell proliferation assay and anchorage-independent assay. Pregnane X receptor suppressed significantly HT29 xenograft tumour growth in nude mice compared with control (310±6.2 *vs* 120±6 mg, *P*<0.01). Immunohistochemistry and immunofluorescence analysis of Ki-67 on excised xenograft tumour tissues showed that PXR inhibited cancer cell proliferation. Furthermore, expressions of PXR and Ki-67 were mutually exclusive. The flow cytometry analysis indicated that PXR caused G_0_/G_1_ cell-cycle arrest. p21^WAF1/CIP1^ expression was markedly elevated whereas E2F1 expression was inhibited by PXR.

**Conclusion:**

PXR inhibits the proliferation and tumourigenicity of colon cancer cells by controlling cell cycle at G_0_/G_1_ cell phase by regulating p21^WAF1/CIP1^ and E2F/Rb pathways.

Pregnane X receptor (PXR), which is also known as the steroid and xenobiotic receptor, was identified as a nuclear receptor that regulates the metabolism and disposition of various xenobiotics and endobiotics (Bertilsson *et al*, 1998; Blumberg *et al*, 1998; Lehmann *et al*, 1998). PXR is a ligand-dependent transcription factor with agonists and antagonists encompassing wide range of structurally diverse endogenous and exogenous compounds. The PXR ligands include more than 50% of clinical drugs/therapeutics, various environmental contaminants such as polyhalogenated and polycyclic aromatic hydrocarbons as well as endogenous substances such as secondary bile acids and steroids. The xenosensor and effector functions of PXR are carried out through coordinately regulating phase I, II and III detoxifying enzymes and membrane-bound transporters (Handschin and Meyer, 2003).

Interestingly, recent results from several laboratories indicate that physiological functions of PXR are not limited to regulation of xenobiotic metabolism. Novel physiological functions of PXR have been discovered including regulation of NF-*κ*B-mediated inflammatory pathway, vitamin D metabolism, cholesterol and lipid homoeostasis, energy homoeostasis, apoptosis as well as cell proliferation (reviewed in Zhou *et al*, 2009). These new results suggest multitudes of PXR functions and potential use of PXR as a therapeutic target for treating certain diseases such as inflammatory bowl disease (IBD), cholestasis and hyperlipidemia in which PXR may influence the disease development.

It is especially noteworthy that several aspects of novel physiological functions of PXR are critical for carcinogenesis and cancer therapy. For example, our recent results suggest that PXR is important in preventing carcinogen-induced DNA damages (Naspinski *et al*, 2008). We and others have found cross talk between PXR-regulated pathway and NF-*κ*B-regulated pathways (Gu *et al*, 2006; Zhou *et al*, 2006). These interactions create a ‘checks and balances’ relationship between pathways, which may have a role in some pathological processes such as IBD, which is a significant cancer risk factor. Indeed, it has been found that *pxr*-null mutant mice showed increased expression of NF-*κ*B target genes and marked intestinal inflammation (Zhou *et al*, 2006) reminiscent of that seen in humans with inflammatory bowel diseases such as the celiac disease (Kagnoff, 2005). Consistent with this PXR-NF-*κ*B mutual repressive cross talk, it has been observed that activation of PXR by the agonist pregnenolone-16*α*-carbonitrile (PCN) significantly reduced dextran sulphate sodium-induced IBD in mouse model (Shah *et al*, 2007). The protective effects of PCN were not seen in *pxr*-null mice indicating the pivotal role of PXR in controlling the chemical-induced inflammation in the intestine. It has also been reported that mouse expressing constitutively active PXR (VP16-PXR) showed significantly reduced damage to the intestinal epithelium from carcinogen or toxic chemicals (Sonoda *et al*, 2002).

Recent studies implicated PXR in regulation of cell proliferation. It was found that PXR-null mice showed impaired hepatocyte proliferation after partial hepatectomy suggesting that PXR is required for normal progression of liver regeneration by modulating hepatocyte proliferation (Dai *et al*, 2008).

Collectively, emerging evidence shows the involvement of PXR in regulating carcinogenesis and tumour progression independent of its known role in detoxification and metabolic activation of carcinogens. To analyse these recently appreciated roles of PXR in regulating cancer cell growth, we performed *in vitro* and *in vivo* experiments using cell culture and xenograft tumour assays. These experiments led to the discovery that PXR is directly involved in suppression of tumour growth through functional regulation of cell-cycle progression through induction of *p21*^*WAF1/CIP1*^ and reduction of *E2F1* expression. These observations strongly suggest that PXR possesses tumour suppressor activity that might be used for cancer prevention and therapy.

## Materials and methods

### Human colon cancer samples

A total of 40 cases of human colon cancer and their adjacent normal colon mucosa tissue specimens were obtained from colon cancer patients (27 men and 13 women, age from 52 to 74 years) in the Department of Pathology, First Affiliated Hospital of Guangzhou Medical College, China, between 2007 and 2008. All cancers were medium-differentiated adenocarcinoma. The samples were fixed with 4% buffer neutralised formalin, paraffin-embedded and cut into 4 *μ*m sections. The protocol for the use of human samples was approved by the institutional review board of the hospital.

### Cell culture, plasmids and antibodies

HT29 and HepG2 cells were cultured in DMEM medium (HyClone, Logan, UT, USA) with 10% fetal bovine serum (Atlanta Biologicals, Lawrenceville, GA, USA) and 1% antibiotics and antimycotics (Gibco) and maintained at 37°C in 5% CO_2_. The PXR-mediated luciferase reporter genes were constructed by inserting the xenobiotic response element module and proximal promoter sequences from CYP3A4 into the pGL3Basic vector (Promega, Madison, WI, USA) as described previously (Gu *et al*, 2006). The antibodies used in this study were from the following vendors: PXR (PP-H4417-00; R&D Systems, Minneapolis, MN, USA), p21^WAF1/CIP1^ (M7202; Dako, Denmark), Ki-67 (sc-15402), p53 (sc-6243) and E2F1 (sc-251) were purchased from Santa Cruz Biotechnology (Santa Cruz, CA, USA).

### Stable transfection and luciferase assay

HT29 cells were seeded in the 12-well plates at about 70% confluence and transfected with PXR expression plasmids (named PXR-HT29) or vector control (Vector-HT29) using Lipofectamine (Invitrogen, Carlsbad, CA, USA) according to the manufacturer's protocol. The transfected cells were then selected by neomycin (1 *μ*g ml^−1^) for 15 days. PXR expression was verified by western blot and immunocytochemical staining using antibody against PXR. For luciferase assay, we treated transfected cells in triplicates with either PXR ligand rifampicin (10 *μ*M) or DMSO as the control for 48 h, then collected and assayed for luciferase activity in a luminometer using the Reporter Lysis Buffer System (Promega). PXR was also transfected into HepG2 cells using the same method.

### MTT assay

Cell viability and proliferation were determined by 3-(4,5-dimethylthiazol-2-yl)-2,5 diphenyltetrazolium bromide (MTT) assay. Briefly, HT29 or HepG2 cells with or without PXR transfection were seeded in 48-well plates with 0.2 ml media per well and were grown for 14 days under standard cell culture conditions. The cultured cells were washed with RPMI-1640 without phenol red and incubated with MTT solution (0.5 mg ml^−1^) at 37°C for 3 h to allow MTT to be metabolised. The medium was removed and 1 ml acidic isopropanol was added for dye conversion in each well. The supernatant was transferred into tubes, centrifuged and measured at a wavelength of 570 nm with background subtraction at 650 nm in disposable cuvettes by a spectrophotometer (Beckman, Brea, CA, USA; DU-600).

### Soft agar assay for colony formation assay

The cultured HT29 or HepG2 cells with or without transfected PXR were mixed with culture medium containing 0.25% agarose and plated at a density of 2 × 10^4^ per well on the pre-solidified bottom of 0.5% agarose with the same medium in six-well plates. The plates were incubated at standard cell culture condition up to 3 weeks. The results were recorded by photography and the colonies with a diameter above 100 *μ*m were determined by ImagJ program (NIH, Bethesda, MD, USA).

### Western blot

The cultured cells were scraped off the plates using cell scraper and the samples were boiled in 2 × SDS sample buffer. The proteins were separated by 8% SDS–polyacrylamide gel (PAGE). Proteins on the gel were transferred to nitrocellulose membranes (Bio-Rad, Hercules, CA, USA) and the membranes were blocked with 5% bovine serum albumin in Tris-buffered saline with 0.1% Tween 20 (TBST) (20 mM Tris-HCL (pH 7.6), 137 mM NaCl, 2.68 mM KCl, 0.05% Tween 20), and incubated with appropriate primary antibodies at 37°C for 60 min. Blots were washed three times with TBST, then incubated with a 1 : 2000 dilution of immunoaffinity-purified goat anti-rabbit IgG linked to alkaline phosphatase (AP). Blots were washed three times with TBST and subsequently developed using the western blot AP substrate (Promega).

### Cell cycle analysis with flow cytometry

Cells transfected with PXR or vector were cultured up to 70%, treated with DMSO or rifampicin for 24 h and collected by trypsinisation. Cells were fixed in 70% ethanol for 2 h, followed washing with phosphate-buffered saline (PBS) twice and re-suspended in propidium iodide (20 *μ*g ml^−1^) staining solution containing 1 mg ml^−1^ RNase in 10 mM PBS (pH 7.4) for 30 min at room temperature. Flow cytometry analysis was performed immediately in an FACSCalibur flow cytometer (Becton Dickinson, Franklin Lakes, NJ, USA) with an excitation at 488 nm and an emission at 620 nm. Triplicate experiments were always performed.

### Immunocytochemistry

To confirm the expression of transfected PXR *in situ*, immunocytochemical staining with PXR antibody was performed in cultured cells. PXR-HT29 and Vector-HT29 cells were seeded in the 8-well chamber slides, cultured for 2 days and fixed with 4% neutral buffered formaldehyde solution for 20 min. Microwave-heated antigen retrieval was performed in 0.01 mol l^−1^ citric acid buffer (pH 6.0) for 15 min. Cells were treated with 0.1% Triton X-100 for 10 min, then blocked with normal donkey serum for 30 min and then incubated with PXR mouse monoclonal antibody (R&D Systems) at dilution 1 : 100 overnight at 4°C. After washing with PBS containing 0.1% Tween 20 (PBST), the biotinylated secondary antibody and the streptavidin–biotin complex (Invitrogen) were applied, each for 30 min at room temperature with an interval PBST washing. 3,3′-Diaminobenzidine (Sigma, St Louis, MO, USA) solution (0.4 mg ml^−1^, with 0.003% hydrogen peroxide) was used as a substrate for developing colour. The slides were then counterstained with haematoxylin, dehydrated and mounted with coverslips. The results were visualised on an Olympus (AH-3; Olympus, Tokyo, Japan) microscope equipped with a SPOT INSIGHT COLOR digital camera, and images were obtained using SPOT DIGITAL CAMERA SYSTEMS software (Diagnostic Instruments Inc., Sterling Heights, MI, USA).

### *In vivo* tumourigenesis assay

Female BALB/c nude mice (24, aged 6–8 weeks) were divided into four groups (Workman *et al*, 1998). A total of 7.5 × 10^6^ HT29 cells with transfected *PXR* gene or vector were subcutaneously injected into the nude mice at right flank. All animals received either corn oil or rifampicin (100 mg/kg per day intraperitoneal) treatment from day 6 to day 16. The tumour size was measured by a vernier caliper every 2 days from day 6 to day 16 after cell implantation. The volume was calculated by a formula: *V*=0.5 *a* × *b*^2^, where *a* is the long diameter and *b* the short diameter. The animals were killed on day 16. The tumour was removed from the body, weighed and cut into two pieces. One piece was fixed with 4% neutral buffered formaldehyde solution and another one was frozen in liquid nitrogen for further assays.

### Immunohistochemistry

Immunohistochemical staining was performed in HT29 tumours from nude mice and human colon cancer samples. Paraffin-embedded sections (4 *μ*m thick) were deparaffinised, re-hydrated and microwave-heated for 15 min in 0.01 mol l^−1^ citric acid buffer (pH 6.0) for antigen retrieval. Then, 3% hydrogen peroxide was applied to block endogenous peroxidase activity. After 15 min of blocking with normal serum (Invitrogen), we applied the primary antibody or corresponding control isotype IgG and incubated overnight at 4°C. Slides were washed thrice with PBS, each for 5 min. Slides were incubated with the biotinylated secondary antibody and the streptavidin–biotin complex, each for 30 min and washed three times at room temperature. After rinsing with PBS, the slides were immersed for 10 min in 3,3′-diaminobenzidine (Sigma) solution (0.4 mg ml^−1^, with 0.003% hydrogen peroxide), monitored under microscope and stop the reaction with distilled water, counterstained with haematoxylin, dehydrated, and coverslipped. The following primary antibodies were applied: PXR (PP-H4417-00; R&D Systems), p21^WAF1/CIP1^ (M7202; Dako) and Rb (OP66; Calbiochem, Gibbstown, NJ, USA); Ki-67 (sc-15402), p53 (sc-6243) and E2F1 (sc-251) were purchased from Santa Cruz Biotechnology. The working concentration of all primary antibodies was 2 *μ*g ml^−1^.

### Immunofluorescence double staining

Paraffin-embedded sections were deparaffinised, re-hydrated and microwave-heated for 15 min in 0.01 mol l^−1^ citric buffer (pH 6.0) for antigen retrieval. After blocking with 10% donkey serum (Jackson ImmunoResearch, West Grove, PA, USA) for 30 min, the primary antibody solution containing mouse anti-PXR antibody (1 : 100) and rabbit anti-Ki-67 antibody (1 : 100) or the solution of corresponding isotype control IgGs at the same concentration with primary antibody was applied and incubated overnight at 4°C. Sections were washed with PBS for three times, each for 5 min. The secondary antibody solution containing donkey anti-mouse antibody conjugated with orange-red fluorescent AF568 and donkey anti-rabbit antibody conjugated with green fluorescent AF488 (both from Invitrogen and at dilution of 1 : 1000) was applied in dark for 30 min. Slides were washed three times with PBS and mounted with aquatic medium contained DAPI.

### TUNEL assay

Terminal deoxyribonucleotide transferase-mediated nick-end labelling (TUNEL) staining was performed using the *In situ* Cell Death Detection kit (Roche Applied Science, Indianapolis, IN, USA) following the instructions of the manufacturer. Briefly, 4-*μ*m-thick formalin-fixed, paraffin-embedded tissue sections were deparaffinised and re-hydrated. Endogenous peroxidase activity was quenched by hydrogen peroxide and tissue protein was hydrolysed with proteinase K. Positive control are sections treated with DNase I 1000 U ml^−1^. Negative control sections are incubated with label solution (without terminal deoxynucleotidyl transferase enzyme). All other sections were incubated with TUNEL reaction mixture (fluorescein-labelled nucleotides) at 37°C for 1 h in a humid chamber, incubated with converter-POD solution (anti-fluorescein antibody conjugated with POD) for 30 min at 37°C, treated with DAB and counterstained with haematoxylin.

### Quantitative measurement

The quantitative analysis of immunohistochemical staining with nuclear positive was performed by PhotoShop and ImageJ (NIH) programs and 10–15 photos per sample were taken randomly. We chose the positive staining by PhotoShop Color Range and saved it as the criterion for all samples. The chosen area was filled with white colour and the rest with black colour. Then the function of Analyze Particle in ImageJ Program was applied to count the number of positive nuclei.

### Statistics

All data were analysed by comparing means with one-way ANOVA method using SPSS (Chicago, IL, USA; version 11.5.0). An additional Duncan's *post hoc* test was followed for the results of luciferase activity and cell-cycle assay. Data are shown as mean±s.e.m. and *P*<0.05 denotes statistically significant difference.

## Results

### Loss of PXR in human colon cancers and restoration of PXR by stable transfection of *PXR* gene in HT29 cells

**Figure 1:**
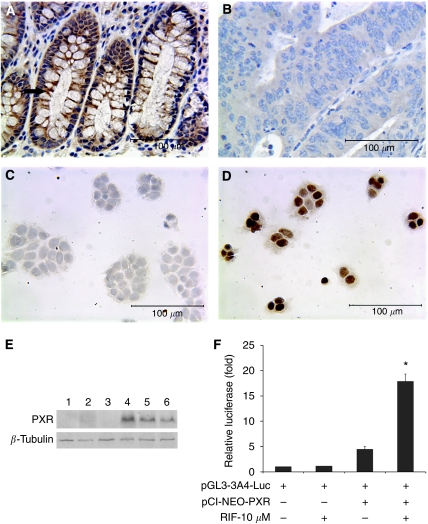
PXR expression is lost in human colon cancer and restored in HT29 cells by stable transfection. The normal human colon mucosae express PXR in the cytoplasm (indicated by arrow), which was lost or greatly diminished in colon cancer cells as determined by immunohistochemistry (**A** and **B**). The expression of PXR was restored by stable transfection of *PXR* gene (compare parental HT29 cells, **C** and HT29 cells stably transfected with PXR, **D**) and expression was confirmed by western blot analysis (**E**, lanes 1, 2, 3 are vector-HT29 and lanes 4, 5, 6 are PXR-HT29) and transcriptional activity of PXR was confirmed by luciferase reporter gene assay in HT29 cells co-transfected with PXR and PXR-driven luciferase reporter gene. The luciferase reporter gene was significantly induced (*P*<0.01) in PXR ligand rifampicin-treated group (^*^indicated) compared with DMSO vehicle control (**F**).

To analyse the role of PXR in colon carcinogenesis, we surveyed the *PXR* gene expression in 30 human colon cancer samples with adjacent normal colon mucosa as control using immunohistochemistry. The normal epithelial cells of 73.3% (22 out of 30) cases showed weak to medium PXR expression (Figure 1A) but 80% (24 out of 30) of cancer samples showed negative staining (Figure 1B).

In an earlier study, we noticed that in many cell lines derived from GI tract, such as HepG2 and HT29 cells, expression of PXR is lost (Xie *et al*, 2009). To investigate the effects of PXR on tumour cell growth, we restored PXR expression through transfection of human PXR into the HT29 colon cancer cell line. The PXR expression levels in the transfected HT29 cells were confirmed by immunocytochemical staining (Figure 1D) and western blot (Figure 1E). The gene regulatory function of this ectopically expressed PXR was tested with co-transfection of a PXR-mediated luciferase reporter gene. Luciferase activity was induced (4.5-fold, *P*<0.05) after transfection with PXR, and the PXR ligand rifampicin markedly increased the luciferase response (15.7-fold, Figure 1F), suggesting that the transfected PXR restored its normal transcriptional properties.

### PXR inhibits proliferation and colony formation of HT29 and HepG2 cells

**Figure 2:**
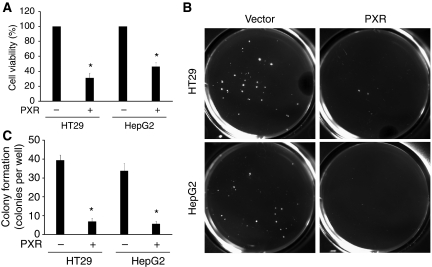
PXR restoration inhibited cancer cell proliferation and colony formation in anchorage-independent culture. MTT assays showed that the viable cells were inhibited significantly in PXR-HT29 and PXR-HepG2 cells comparing to vector control cells (^*^, *P*'s<0.01) at day 14 time point after cells seeding on the plates (**A**). For soft agar assay, the results were recorded by microphotography and the colonies with a diameter above 100 *μ*m were counted by ImageJ program (**B**), and the number of colonies was markedly decreased in both PXR-HT29 and PXR-HepG2 cells compared with vector control cells. PXR stands for PXR-transfected HT29 or HepG2. In (**C**), ^*^, *P*'s<0.01.

Upon expression of PXR through stable transfection of HT29 and HepG2, we noticed that PXR expression inhibited cell growth. To further investigate the PXR-regulated cell proliferation, we performed MTT assays to quantify the difference of cell growth and proliferation in colon cancer cell line HT29 and liver cancer cell line HepG2. The results showed that the cell proliferation was inhibited significantly (68.7% inhibition, *P*<0.01) in PXR-HT29 group compared with Vector-HT29 group and 53.6% (*P*<0.01) reduction in PXR-HepG2 cells compared with Vector-HepG2 cells at day 8 after cells seeding (Figure 2A).

To further characterise the growth-inhibiting property of PXR, we performed anchorage-independent growth assays in soft agar (Figure 2B). The presence of PXR significantly reduced colony formation in HT29 cells (39.3±2.7 *vs* 7.0±1.5 colonies per well, *P*<0.01) and HepG2 (33.7±3.8 *vs* 5.7±1.5, *P*<0.01) 3 weeks after cell seeding on the plates (Figure 2C).

### PXR inhibits xenograft tumour growth

**Figure 3:**
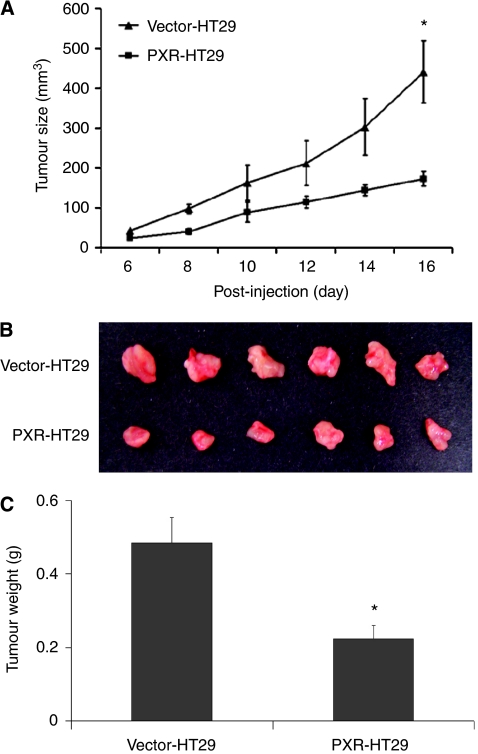
PXR inhibits growth of tumour xenograft in nude mice. PXR-HT29 or Vector-HT29 cells were injected subcutaneously into nude mice at concentration of 7.5 × 10^6^ per injection. The tumour size was measured every 2 days. The tumour growth of PXR-HT29 group was significantly inhibited in the xenograft carrying PXR than that of control group at 16 days after initial injection (**A**, ^*^P<0.01, *n*=12). The tumours were then removed from the nude mice and imaged (**B**) and the final weights were significantly different between PXR-HT29 and Vector-HT29 groups (**C**, ^*^P<0.01, *n*=12).

To further analyse the tumour inhibitory effects of PXR, we implanted HT29 cells transfected with PXR or vector into nude mice. The tumour volumes were measured every other day. The tumour volume calculated by both long diameter and short diameter in PXR-HT29 group was significantly smaller than that in Vector-HT29 group (441±78 *vs* 173±18 mm^3^, *P*<0.05) at day 16. The growth rate of the tumour xenografts was significantly lower than the vector control group (Figure 3A). The tumours were removed from mice on the termination of the experiments and showed a readily apparent difference in size between Vector-HT29 group and PXR-HT29 group (Figure 3B). The final tumour weight of the xenografts was significantly different between two groups (310±6.2 *vs* 120±6 mg, *P*<0.01; Figure 3C).

### PXR inhibits cell proliferation as determined by Ki-67 staining but does not induce apoptosis in HT29 xenograft tumours

**Figure 4:**
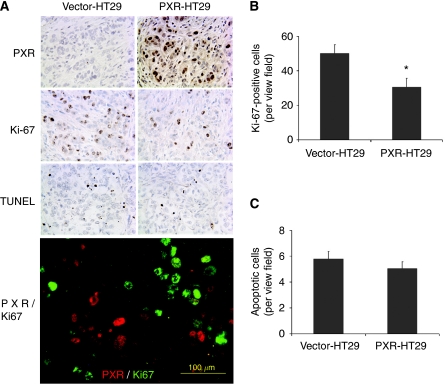
PXR inhibits cell proliferation. The xenograft tumours removed from nude mice were analysed by immunohistochemistry for expression of PXR and Ki-67. TUNEL assay was also performed to determine the level of apoptosis. Ki-67-positive cells were markedly decreased in PXR-HT29 xenograft tumour tissues in comparison with the control (**A** and **B**). There were no significant differences in apoptotic levels between PXR-HT29 and control as determined by TUNEL assay (**A** and **C**). Immunofluorescence double staining of PXR-HT29 xenograft tumour tissues showed a mutually exclusive distributing pattern of PXR and Ki-67 (**A**, bottom) further confirming the anti-proliferative effect of PXR. ^*^P<0.01.

To determine the mechanism by which PXR inhibited the tumour growth in nude mice, we measured cell proliferation and apoptosis by immunohistochemistry, immunofluorescence double staining and TUNEL assay in the excised xenografts. Robust nuclear expression of PXR protein was retained in most of the tumour cells (>50%) from the PXR-HT29 xenografts (Figure 4). Cells positive for the proliferation marker Ki-67 were noticeably reduced in PXR-HT29 xenografts (Figure 4A). The number of Ki-67-positive cells was quantified using the ImageJ program, and significantly low number of Ki-67-positive cells were found in the PXR-HT29 xenografts (50.13±4.96 *vs* 30.47±5.19, *P*<0.01; Figure 4B). To determine the level of apoptosis in the xenograft, we performed TUNEL assay. There were no significant differences in the PXR-HT29 and vector control xenograft (5.79±0.57 *vs* 5.03±0.53 cells per field, *P*>0.05; Figure 4A and C), suggesting that the reduction of tumour sizes was not caused by apoptosis. Interestingly, results of PXR and Ki-67 immunofluorescence double staining indicate that the expression levels of PXR and Ki-67 are mutually exclusive (Figure 4A, bottom panel), strongly suggesting the anti-proliferative function of PXR.

### PXR suppresses cell proliferation by induction of G_0_/G_1_ cell-cycle arrest

**Figure 5:**
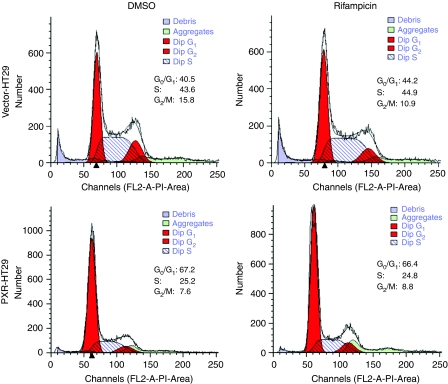
PXR suppresses cell proliferation by causing G_0_/G_1_ arrest in cell cycle. PXR-HT29 and Vector-HT29 cells were stained with propidium iodide and analysed by flow cytometry. The percentage of cell population in G_0_/G_1_ phase was significantly higher in PXR-HT29 group (lower panel) than in Vector-HT29 group (67.2±1.9 *vs* 40.5±1.4%, *P*<0.01) suggesting G_0_/G_1_ arrest.

To determine the cell phase of HT29 being regulated by PXR, we stained the cells with propidium iodide and analysed by flow cytometry. The results indicated that the percentage of cells at G_0_/G_1_ phase was significantly higher in PXR-HT29 cells than in Vector-HT29 cells (67.2±1.9 *vs* 40.5±1.4%, *P*<0.01) and the percentage of cell population at S phase and G_2_/M phase was significantly lower in PXR-HT29 cells than in Vector-HT29 cells (25.2±0.6 *vs* 43.6±1.2%, *P*<0.01 and 7.6±0.6 *vs* 15±2.5%, *P*<0.01, respectively), suggesting that induction of G_0_/G_1_ cell-cycle arrest was the primary mechanism by which PXR inhibits proliferation in HT29 cells (Figure 5). This experiment was conducted with control and PXR ligand, and cell-cycle suppressive effects of PXR seem to be ligand independent.

### PXR upregulates p21^WAF1/CIP1^ expression and inhibits E2F1 expression

**Figure 6:**
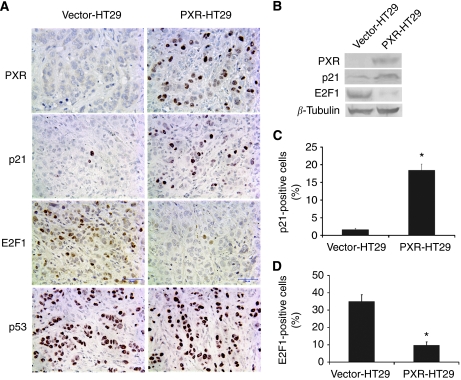
PXR expression increases p21^WAF1/CIP1^ expression and suppresses E2F1 expression. HT29 tumour tissues from nude mice were immunohistochemically stained with PXR, E2F1 and p53, and all show nuclear localisation (**A**). Western blot further confirmed the expression of PXR, p21^WAF1/CIP1^ and E2F1 with cultured cells between two groups (**B**). The cells with nuclear positive staining were counted by PhotoShop and ImageJ programs and the data were statistically analysed by one-way ANOVA test. The number of p21^WAF1/CIP1^-positive cells is higher in PXR-HT29 group than in vector control group (**C**, ^*^P<0.01). However, the percentage of E2F1-positive cells is markedly lower in PXR-HT29 group than Vector-HT29 group (**D**, ^*^P<0.01).

p21^WAF1/CIP1^ has been found to have a crucial regulatory role in cell-cycle control by inhibiting CDK4 and preserving the Rb/E2F complex. Immunohistochemistry staining of xenograft tumours showed that p21^WAF1/CIP1^ expression was remarkably higher in PXR-HT29 tumours than in Vector-HT29 tumours (Figure 6A and C), but p53 expression appeared to be the same in the two groups most likely due to p53 mutation in HT29 cells.

E2F/Rb signal transduction is one of the most important pathways for cell growth progression in the G_1_ phase of the cell cycle. When Rb is phosphorylated by cyclin D–CDK4 complexes E2F is released from the Rb–E2F complex and promotes cell-cycle progression. From immunohistochemistry staining, we found E2F1 expression was significantly lower in PXR-HT29 tumours compared with Vector-HT29 tumours (Figure 6A and D). Western blot analysis further confirmed the differential expression of E2F1 in the xenograft tumours from both groups (Figure 6B). The most well-known regulator of p21^WAF1/CIP1^, p53, is mutated in HT29 cancer cell line and the amount of the transcriptionally inactive p53 found in these cells accumulates in the nucleus. PXR expression did not change p53 expression in these xenograft tumours.

## Discussion

Although PXR has been recognised for its involvement in xenobiotic/drug metabolism through its role as a ligand-dependent transcription factor in the regulation of detoxifying enzymes such as CYP3A4, UGT1A1, GST and transporters (Handschin and Meyer, 2003), recent research reveals unexpected novel physiological functions, many of which seem to be rooted at its fundamental role as the sensor and effector guarding the organism against mutagenic insults from xenobiotics and endobiotics. Interestingly, in addition to reducing mutagenic DNA damages, the physiological functions of PXR may have further evolved to protect cells from unregulated proliferation, which is a critical step for tumourigenesis. In earlier studies, we observed that many cancer cells lines derived from GI tract, such as HepG2, HT29 and HCT116, showed loss of PXR expression (Xie *et al*, 2009; data not shown). Upon ectopic expression of PXR through transfection, the PXR function could be restored and cells showed significant reduced proliferation, suggesting its role in controlling tumour cell proliferation. It has been reported that certain polymorphisms of *PXR* were identified to be strongly associated with the susceptibility to IBD (Dring *et al*, 2006), which is a significant risk factor for colon cancer. In patients with IBD, decreased expressions of PXR and its target genes have been observed (Langmann *et al*, 2004; Martinez *et al*, 2007). On the basis of collective evidence, we hypothesised that PXR has tumour suppressor activity and investigated the effects of PXR expression in PXR-negative colon cancer cells *in vitro* and *in vivo* (Workman *et al*, 1998).

In this study, we have shown that transfected PXR significantly suppressed HT29 cancer cell proliferation, anchorage-independent growth and tumourigenicity in xenograft assays. In tumour xenograft model, the PXR expression was retained in the HT29 cells 2 weeks after xenograft transplantation into nude mice and the tumour size was markedly suppressed by PXR. Interestingly, results of immunofluorescence double staining suggest that PXR and Ki-67 expressions are mutually exclusive (Figure 4), suggesting at cellular level the presence of PXR is inhibitory for the colon cancer cell growth. At organ/tissue levels however, PXR may regulate the growth of cell/tissues through different mechanisms. Wan and colleagues reported the mouse lacking PXR showed decreased liver regeneration after partial hepatectomy and the PXR-regulated lipid homoeostasis, which fuels the liver regeneration, may have a role in the suppressed liver growth (Dai *et al*, 2008). Verma *et al* (2009) reported that PXR activation induced apoptosis in breast cancer cell line and PXR is anti-proliferative and the effect is mechanistically dependant on the local production of NO and NO-dependent upregulation of p53. As the HT29 cells have mutated p53, the mechanism of PXR-regulated inhibitory effects on HT29 growth may differ from the NO-dependent upregulation of p53 reported. Interestingly, Gupta *et al* (2008) reported that in ovarian cancer cell lines certain PXR target genes such as *CYP3A4* and *UGT1A1* were inducible by rifampicin and the ligand promoted the proliferation of the ovarian cancer cell line in culture and xenograft model. The underlying mechanisms for these differences in the roles of PXR in regulating proliferation and apoptosis of colon, breast and ovarian cancer cells are not clear and warrant further investigation. The important questions that await further investigation are what is the physiological level of PXR expression in various tissues, what are the factors that influence its expression/functions, and ultimately, what is the mechanism by which PXR regulates cell cycle.

To investigate the role of PXR in regulation of cell cycle, we first performed flow cytometry analysis of the effects of PXR on the cell cycles and our study results showed that PXR caused G_0_/G_1_ cell-cycle arrest. Cyclin-dependent kinase (CDK) inhibitors p21Cip1/Waf are important as the negative regulator of cell-cycle progression (Gartel and Radhakrishnan, 2005). Normally p21^WAF1/CIP1^ has an inhibitory role regarding cyclin D–CDK4 complex phosphorylation of Rb and is regulated principally by the tumour suppressor p53. The *p53* gene is mutated in HT29 cell line and is transcriptionally inactive. Our study results showed that p21^WAF1/CIP1^ expression is almost completely absent in Vector-HT29 cancer cells but highly expressed in PXR-HT29 cells determined by immunohistochemistry analysis and western blot analysis (Figure 6A–C). Interestingly, the expression of E2F1 was negatively correlated with the p21 expression, suggesting its expression is downregulated by the presence of PXR. It has been shown that control of the p53–p21^CIP1^ axis by E2F family genes is essential for G_1_/S progression and cellular transformation (Sharma *et al*, 2006). It is possible that the negative interaction between p21 and E2F1 was lost in certain colon cancer cells and restored on PXR expression, the mechanism of reduction of E2F1 remains to be investigated. Comparison of the apoptotic cells in the xenograft tumour tissues by TUNNEL staining shows no significant difference between the PXR-HT29 and parental HT29 tumours. However, flow cytometry analysis with the Annexin V antibody staining in cell culture showed slight increases in apoptosis in PXR-HT29 cells (data not shown). Therefore, both cell-cycle regulation and apoptosis may have a role in PXR-regulated suppression of colon cancer growth, however, overall our study results shows that the regulation of cell-cycle appears to be a primary mechanism by which PXR inhibits tumour growth.

In conclusion, our study results suggest that PXR has a novel anti-proliferative function *in vitro* and *in vivo*, beyond its known role in control of metabolism. This function appears to be exerted at least in part through p21^WAF1/CIP1^ control of the E2F/Rb pathway in HT29 cells. These novel results suggest potential applications for new therapies for colon cancer treatment based on modulation of PXR function.
